# Can the neutrophil-to-lymphocyte ratio be used as an early marker of small fetuses for gestational age? A prospective study

**DOI:** 10.3389/fmed.2024.1439716

**Published:** 2024-08-14

**Authors:** David Salomon, Arrigo Fruscalzo, Michel Boulvain, Anis Feki, Nordine Ben Ali

**Affiliations:** Department of Obstetrics and Gynecology, Cantonal Hospital, University of Fribourg, Fribourg, Switzerland

**Keywords:** neutrophil-to-lymphocyte ratio, small for gestational age, fetal growth restriction, uteroplacental failure, hypoxia, inflammatory response

## Abstract

**Background:**

Small-for-gestational-age (SGA) fetuses are at increased risk of mortality and morbidity, and less than 30% will be detected by any ultrasound scan within 4 weeks before delivery. Our aim was to evaluate the relationship between neutrophil/lymphocyte ratio (NLR) in the first trimester of pregnancy and SGA fetuses.

**Method:**

We performed a prospective study between June 2021 and August 2022, to evaluate the relationship between the neutrophil to lymphocyte ratio in maternal blood in the first trimester of pregnancy, with the birth of an SGA fetus. One hundred ninety-four participants with singleton pregnancies between 11 + 1 and 13 + 6 weeks of gestation were recruited. Pregnancies affected with diagnosed fetal chromosomal abnormalities, or chronic pathologies were excluded. SGA was defined as birthweight less than the 10th centile (*N* = 42) and severe SGA as birthweight less than the 3rd centile for gestation (*N* = 10) according to a locally derived descriptive charts. The NLR value measured in the first trimester was compared between these two groups and controls.

**Results:**

We found no statistically significant difference in NLR, (3.5 +/−1.2 vs. 3.4+/−1.2, *p*-value of 0.78) when comparing the SGA less than the 10th centile group to the control group. NLR was also not different between severe SGA and controls (3.6+/−1.4 vs. 3.4+/−1.2 *p*-value of 0.78).

**Conclusion:**

We found no association between first-trimester NLR ratio and SGA.

## Introduction

Worldwide, the estimated rate of small-for-gestational-age (SGA) infants is around 16%, ranging from an average of 7% in developed countries to 41.5% in developing countries, mainly in South Asia (1, 2), and 22% of neonatal deaths are directly linked to SGA (3).

Currently, screening for an SGA fetus is based on ultrasound estimation of fetal weight, using the Hadlock formula (4). An SGA fetus is defined by the International Society of Ultrasound in Obstetrics and Gynecology (ISUOG) as an estimate of fetal weight (EFW) or abdominal circumference (AC) below the 10th percentile (< P10) of expected size, according to a population-based reference range (5). However, it is important to distinguish between constitutionally small but healthy fetuses and fetal growth restriction (FGR), which fail to reach their growth potential due to impaired placental function, with a greatly increased risk of adverse perinatal outcome (5).

In 2016, a panel of international experts reached a Delphi consensus (6) that differentiated between these two entities, defining FGR as EFW or abdominal circumference less than the 3^rd^ centile or EFW or AC less than the 10th centile combined with abnormal Doppler findings or a fall in growth centiles.

To date, detecting fetal growth anomalies is a challenge, and in practice, over 50% of cases of growth retardation are eventually discovered and diagnosed at birth, even in high-income countries (7).

Tape measurement of symphysis-fundal height (SFH) is simple, inexpensive and widely used during antenatal care. Compared with abdominal palpation, there is insufficient evidence to determine whether SFH measurement is effective in detecting intrauterine growth restriction (8).

The most accurate screening method for a SGA fetus still remain the use of an ultrasound estimation of fetal weight, using the Hadlock formula (4), as late as possible in the third trimester. However, ultrasound weight estimation is approximate and most developing and developed countries do no perform 3^rd^ trimester ultrasound routinely and when it is performed, less than 30% will be detected by ultrasound within 4 weeks of delivery (9).

For reasons of limited human and financial resources, selection according to risk factors may be more appropriate for selecting patients who should undergo a 3rd trimester ultrasound.

It has been shown that the rate of SGA detection based on risk factors detects around 40% of SGA (10). On the other hand, systematic ultrasound in the third trimester increases detection sensitivity from 20% to almost 60% of SGA cases (11). This partly explains the high rate of non-discovery of SGA during pregnancy.

A child with SGA below the 10th percentile has a threefold higher risk of morbidity and mortality than a child of appropriate weight (12).

It is therefore vital to be able to detect these fetuses in order to ensure adequate follow-up, but also to be able to act on prevention in order to improve outcome as early as possible in pregnancy. For example, it has been shown that the administration of 150 mg/day of acetylsalicylic acid (ASA) before 16 weeks’ gestation reduces the incidence of SGA less than the 10th centile by around 40% in the case of births before 37 weeks’ gestation, and by up to 70% in the case of births before 32 weeks’ gestation (13).

Fetal growth depends on many factors, which may be maternal, placental or fetal. If one or more of these factors is altered, the mechanisms regulating growth will be impaired. In 60% of fetuses with growth restriction, no cause is identified (14). When the etiology is not idiopathic, genetic, malformative or infectious, placental insufficiency is the most frequently identified cause (15).

Maternal vascular malperfusion (MVM) is the result of insufficient spiral arterial flow due to defective remodeling of the corresponding arteries during pregnancy. It is between 10 and 12 weeks of pregnancy that the remodeling of the spiral arterioles takes place, and therefore maternal arterial circulation to the intervillous space is fully established (16). If this process is defective, it can lead to a reduction in blood flow, resulting in hypoxic–ischemic lesions (17).

Hypoxia results in a persistent inflammatory response mediated by neutrophils, an essential component of the innate immune system, which are the first cells recruited to inflammatory sites attracted by various chemokines. Neutrophils, with their pro-inflammatory activity and oxidative stress, contribute to the recruitment and modulation of T-cell activity, which contributes to tissue damage and alteration of local vascular tissue (18, 19). This will lead to a reduction in the volume of blood and surface area for mother-fetus exchange, affecting placental transport and potentially restricting growth.

Based on this observation, many teams have taken a close interest in the Neutrophil-to-Lymphocyte ratio, calculated by dividing the absolute number of neutrophils by the absolute number of lymphocytes. It is a hematological marker associated with a pro-inflammatory state, simple to perform, inexpensive, can be carried out anywhere in the world, and is used as a means of detecting various pathologies.

Our aim was to evaluate the relationship between neutrophil/lymphocyte ratio (NLR) in the first trimester of pregnancy and SGA fetuses.

## Materials and methods

We conducted a prospective unmatched case–control study in the gynecology and obstetrics department of the Cantonal Hospital of Fribourg (Switzerland) from 01/06/2021 to 31/08/2022.

The inclusion criteria were all women aged >18 years with a single pregnancy who had undergone a first-trimester examination (ultrasound and blood test including at least a complete blood count (CBC) as well as PAPP-A and hCG) in our department.

Exclusion criteria included patients who were minors, multiple pregnancies, genetic pathologies such as Down’s syndrome (trisomy 21), Patau’s syndrome (trisomy 13) and Edwards’ syndrome (trisomy 18), and fetal malformations detected during the first-trimester ultrasound. Patients affected by pathologies modifying the hematological lineage such as Lupus erythematosus, lymphoma, leukemia, human immunodeficiency virus (HIV), patients on long-term corticosteroids and those suffering from inflammatory rheumatic diseases such as ankylosing spondylitis were also excluded. We also excluded patients taking treatments that affect placentation, such as aspirin.

The study was approved by the ethics committee (CER-VD; Project-ID: 2021–00472) and was conducted in accordance with the protocol of the Declaration of Helsinki, the Law on Research Involving Human Subjects (LRH) and the Swiss Ordinance on Research Involving Human Subjects (ORH). Each participant in this study read and signed the informed consent form prior to inclusion.

Patients underwent venous blood sampling between 11 0/7 and 13 6/7 weeks’ gestation. All 3 mL blood samples with EDTA were processed and analyzed by the hospital laboratory (international accreditation ISO/CEI 17025:2017, ISO 15189: 2012.).

Fetal biometry was performed between 32 and 34 weeks of amenorrhea according to our pregnancy follow-up protocol, and EFW was calculated using the Hadlock formula (4).

In pregnancies with suspected SGA, ultrasound evaluation, including Doppler evaluation, was performed at regular intervals. Induction of labor was performed for standard maternal or fetal obstetrical indications.

During labor, cardiotocographic monitoring was performed and interpreted according to the 3-level classification of the FIGO 2015 system (normal, suspicious or pathological).

After delivery, medical staff weighed the newborn within the first 24 h of life, and the weight was compared with the growth curve according to a locally derived descriptive charts used in our institution (20). SGA was defined as a birth weight below the 10th percentile, and severe SGA as a birth weight below 3rd percentile (<P3) also specified as an autonomous criterion for the definition of a FGR under the various guidelines (21, 22). Newborns with a birth weight of the 10th percentile or above were considered appropriate for gestational age (AGA).

Placental weight was measured using an electronic balance within 1 h of placental expulsion. The placenta was weighed with membranes and cord preserved and without prior fixation.

### Statistical analysis

Data were analyzed using Stata 17.0 software (StataCorp LLC, Texas, United States). Results are expressed as mean +/− standard deviation (minimum-maximum) or number and percentage.

Based on expected first-trimester NLR values [AGA 3.53 (CI 1.76; 6.73) vs. SGA 2.9 (+/−0.96)] (23, 24) using a case–control ratio of 1:3, to obtain a study power of >0.80 with α = 0.05 we estimated a minimum sample size of patients for cases and controls of 41 and 123, respectively. To compare demographic variables and the outcomes between the SGA and the control population, we used a Student’s T-test for continuous variables and a Fisher’s exact test for categorical variables. Differences were considered significant if the *p*-value was <0.05. Results were adjusted for smoking and history of SGA with a logistic regression.

## Results

We included a total of 194 women, and. Forty-two women (21.6%) had a newborn weighing less than 10th percentile and, of these, 10 had a birth weight below the 3rd percentile representing 23.8% of total SGA.

The demographics of the participants are summarized in Table 1. There was no significant difference in age, with a median age of 28.1 +/− 4.7 in the SGA group versus 28.4 +/− 5.0 in the AGA group. In terms of gestity and parity, the results were also similar in the 2 groups, i.e., 2.5+/−1.2 vs. 2.4+/−1.5 for gestity and 0.9+/−1.1 vs. 0.8+/−0.9 for parity. In terms of origin, we also found similarities between the two groups, with an unsurprising predominance of Caucasian origin 80.9% in the SGA group vs. 84.2% in the control group.

**Table 1:** Patient demographics of women in the small for gestational age (SGA) groups, compared to the appropriate for gestational age (AGA) group.

Characteristics	AGA (*n* = 152)	SGA < P10 (*n* = 42)	*p*- value	SGA < P3 (*n* = 10)	*p*- value
Age (years)	28.4 (+/−5.0)	28.1 (+/−4.7)	0.44	27.9 (+/−4.1)	0.39
Ethnic origin			0.75		0.68
Caucasian	128 (84.2%)	34 (80.9%)		8 (80.0%)	
African	19 (12.5%)	7 (16.7%)		2 (20.0%)	
Asian	5 (3.3%)	1 (2.4%)		0	
Nulliparous women	65 (42.7%)	19 (45.2%)	0.77	5 (50.0%)	0.65
Past history of SGA	0	3 (7.1%)	<0.001	3 (30.0%)	<0.001
Pregnancy onset					
ART	7 (4.6%)	0	–	0	–
Medical history					
HTD^a^	8 (5.2%)	3 (7.1%)	0.64	1 (10.0%)	0.52
Diabetes^b^	40 (26.3%)	11 (26.2%)	0.98	2 (20.0%)	0.65
Thyroid disease^c^	13 (8.5%)	2 (4.7%)	0.41	0	**–**
Current smoker	17 (11.2%)	8 (19.0%)	0.17	1 (10%)	0.9
BMI category (kg/m2)			0.33		0.01
<25	66 (50.7%)	23 (60.4%)		6 (66.7%)	
25–30	37 (28.5%)	8 (21.0%)		1 (11.1%)	
>30	27 (20.7%)	7 (18.4%)		2 (22.2%)	

Results expressed as *n* = number (%) percentage or mean (SD) standard deviation if appropriate. ART, assisted reproduction technology. ^a^HTD=Hypertensive disorders; includes chronic hypertension, gestational hypertension, and preeclampsia. ^b^Diabetes includes preexisting and gestational diabetes. ^c^Thyroid disease includes hypothyroidism or hyperthyroidism with or without autoimmune causes. BMI, Body mass index. *p* values calculated by a student’s *t*-test or the Fisher’s exact test.

In terms of couples requiring assisted reproduction, only 4.6% of couples in the AGA group required pregnancy assistance, and no couples in the SGA group required assisted reproduction.

Concerning medical history, there were no statistically significant differences either in terms of the spectrum of hypertensive diseases, or thyroid pathology, or in terms of pre-existing or gestational diabetes. Regarding weight, patients had identical body mass index (BMI) in both groups, with a predominance of BMI in the <25 kg/m2.

The proportion of smokers was significantly higher in the SGA group (19.0% vs. 11.2%, *p* value 0.17), but not in the severe SGA group (10%, *p* value 0.90). We also found 7.1% of women with a history of newborn below the 10th percentile in the SGA group, versus none in the control group (*p* value <0.001), with an even higher proportion in the severe SGA group (30.0%, *p* value <0.001).

The results concerning pregnancy outcome characteristics are presented in Table 2. We found no significant difference in amniotic fluid abnormalities, preterm birth (<37 weeks’ gestation) or labor induction, with the exception of preterm birth and labor induction in the severe SGA group, but this difference was not significant (30.0%, *p* value 0.01, respectively, and 30.0%, *p* value 0.20). Of our 42 SGA patients, five (11.9%) required induction of labor due to ultrasound criteria or altered fetal status between 36 to 38 2/7 weeks’ amenorrhea. Women with SGA newborns underwent more non-invasive prenatal tests (NIPT) (28.6 and 30.0% for the SGA below the 3rd percentile group) than in the AGA group (11.3%) (*p* value 0.005 and P 0.008 respectively).

**Table 2:** Pregnancy characteristics of the SGA groups compared to the AGA group.

Characteristics	AGA (*n* = 152)	SGA < P10 (*n* = 42)	*p*-value	SGA < P3 (*n* = 10)	*p*-value
NIPT performed	17 (11.3%)	12 (28.6%)	0.005	3 (30.0%)	0.008
Corticosteroïds for lung maturation	5 (3.3%)	0	–	0	**–**
Umbilical Doppler abnormality	0	1 (2.4%)	0.12	1 (10.0%)	<0.001
Oligohydramnios	1 (0.6%)	1 (2.4%)	0.59	1 (10.0%)	0.03
Preterm birth (< 37 weeks)	11 (7.2%)	5 (11.9%)	0.33	3 (30.0%)	0.01
Induction of labor	53 (34.9%)	15 (35.7%)	0.24	3 (30.0%)	0.20

Results expressed in *n* = number (%) percentage. SGA, small for gestational age; AGA, appropriate for gestational age; NIPT, Non-invasive prenatal testing; ASA, acetylsalicylic acid. *p* values calculated by the Fisher’s exact test.

Delivery results and neonatal outcomes are summarized in Table 3. Mean gestational age at delivery did not differ between the two groups.

**Table 3:** Course of delivery for the SGA group compared to the AGA group.

Characteristics	AGA (*n* = 152)	SGA < P10 (*n* = 42)	*p*- value	SGA < P3 (*n* = 10)	*p*- value
Gestational age at delivery	39.6 (+/−1.6)	39.7 (+/−1.7)	0.65	39.2 (+/−1.3)	0.57
Mode of delivery			0.09		0.60
Spontaneous	75 (49.3%)	28 (66.6%)		5 (50.0%)	
Instrumental	33 (21.7%)	4 (9.5%)		1 (10.0%)	
C-section	44 (28.9%)	10 (23.8%)		4 (40.0%)	
Gender			0.09		0.02
Female	65 (42.7%)	24 (57.1%)		8 (80.0%)	
Male	87 (57.2%)	18 (42.8%)		2 (20.0%)	
CTG			0.58		0.89
Suspicious	23 (15.1%)	9 (21.4%)		2 (20.0%)	
Pathological	13 (8.5%)	4 (9.5%)		1 (10.0%)	
APGAR <7			0.12		0.20
1 min	9 (5.9%)	6 (14.3%)		2 (20.0%)	
5 min	4 (2.6%)	0	–	0	–
Umbilical cord pH			0.89		0.67
>7.20	118 (78.6%)	33 (80.5%)		7 (77.7%)	
7.20–7.15	18 (12.0%)	4 (9.7%)		2 (22.2%)	
7.15–7.10	8 (5.3%)	3 (7.3%)		0	
<7.10	6 (4.0%)	1 (2.4%)		0	
Placenta weight after delivery	586.5(+/−110.3)	462.4 (+/−92.3)	<0.001	423.5 (+/−93.5)	<0.001
Ratio BW/PW	6.0(+/−1.3)	5.9 (+/−1.4)	0.98	5.5 (+/−0.9)	0.18
Admitted to neonatal unit	21 (13.8%)	6 (14.3%)	0.93	5 (50.0%)	0.003

Results expressed as *n* = number (%) percentage or mean (SD) standard deviation if appropriate. SGA, small for gestational age; AGA, appropriate for gestational age; CTG, cardiotocogram. Ratio BW/PW=Birth weight/Placenta weight. *p* values calculated by a student’s *t*-test or Fisher’s exact test.

In terms of mode of delivery, spontaneous delivery was predominant in the SGA group (66.6% vs. 49.3% for the AGA group), and the number of cesarean sections and instrumentations was lower in the SGA group (23.8% vs. 28.9 and 9.5% vs. 21.7% respectively), although this was not statistically significant.

The cardiotocogram (CTG) during labor was analyzed according to FIGO classification and no statistically significant difference was found between the study populations. The same results were also found for pH levels at birth.

Regarding APGAR below 7 at 1 and 5 min of life, at 1 min of life, 14.3% of newborns had APGAR below 7, versus 5.9% in the AGA group, although this was not statistically significant. At 5 min of life, APGARs below 7 were present only in 2.6% of cases in the AGA group.

In terms of the immediate and short-term fate of these newborns, there was no difference in the rate of hospitalization in the neonatal unit 13.8% for the AGA group versus 14.3% for the SGA below the 10th percentile group (*p* value 0.93), but we found 50.0% hospitalization in the SGA below the 3rd percentile group (p value 0.003).

Finally, in terms of placental weights, we found a mean weight of 586.5 g (+/− 110.3) in the AGA group vs. 462.4 g (+/−92.3) in the SGA group for a *p* value of <0.001, and 423.5 g (+/−93.5) for a p value of <0.001 in the severe SGA group. Concerning the birth weight/placental weight ratio, we found no significant difference between the AGA and SGA groups, respectively 6.0(+/−1.3) vs. 5.9(+/−1.4) p value 0.98, and 5.5(+/−0.9) for a p value 0.18 in the severe SGA group.

The values of the various hematological markers are given in Table 4. We found no statistically significant difference in hematological markers between groups. The mean NLR was 3.4 (+/− 1.2) in the SGA group, 3.6 (+/− 1.4) in the severe SGA group and 3.5 (+/− 1.2) in the AGA group.

**Table 4:** Hematological marker results.

Characteristics	AGA (*n* = 152)	SGA < P10 (*n* = 42)	*p*- value	SGA < P3 (*n* = 10)	*p*- value
Hemoglobin (g/l)	128.7 (+/−10.1)	129.4 (+/−9.5)	0.67	131.2 (+/−8.8)	0.41
Thrombocytes (G/l)	258.0 (+/−62.4)	253.4 (+/−50.2)	0.57	267.2 (+/− 56.1)	0.65
Leukocyte (G/l)	8.9 (+/−2.3)	8.8 (+/−1.9)	0.59	9.1 (+/−1.7)	0.76
Neutrophil (G/l)	6.3 (+/−1.9)	6.1 (+/−1.7)	0.58	6.4 (+/−1.7)	0.79
Lymphocyte (G/l)	1.9 (+/−0.6)	1.9 (+/−0.4)	0.66	1.9 (+/−0.4)	0.88
NLR	3.5 (+/−1.2)	3.4 (+/−1.2)	0.78	3.6 (+/−1.4)	0.78

Results expressed as mean (SD) standard deviation. *n*, number of patients. SGA, small for gestational age; AGA, appropriate for gestational age; NLR, Neutrophil-to-Lymphocyte Ratio. *p* values calculated by a student’s *t*-test.

We ran a combined logistic regression (Table 5) showing adjusted ORs for NLR, smoking and multiparity.

**Table 5:** Logistic regression including risk factors for SGA and NLR.

Characteristics	Odds ratio	[95% CI]	*p*- value
NLR	1.03	[0.77–1.38]	0.82
Tobacco	0.53	[0.21–1.33]	0.18
Multiparity	1.13	[0.56–2.27]	0.71
Baseline OR	3.29	[1.06–10.23]	0.04

NLR, Neutrophil-to-Lymphocyte Ratio; CI, confidence interval. *p* values calculated by a student’s *t*-test.

## Discussion

In our prospective study, we explored the relationship between first-trimester maternal NLR value and SGA and our results do not find a relationship between first-trimester NLR and fetal growth.

The Neutrophil-to-Lymphocyte ratio has been the subject of much research in the field of obstetrics. It has been shown, for example, that the NLR value in the first trimester of pregnancy is higher in patients with preeclampsia than in those without (25).

Another research topic showed a significant increase in NLR during the 1st and 3rd trimester in patients with gestational diabetes mellitus (GDM) compared with women without GDM, suggesting a key role from the early stages of pregnancy in the occurrence of systemic inflammation, which could be a trigger for the onset of GDM (26, 27).

A meta-analysis also showed that NLR can be a predictive tool for spontaneous preterm delivery (28).

Another research topic showed that NLR could be used as a predictor factor in miscarriages, which was demonstrated by a meta-analysis showed that the NLR was significantly higher in the miscarriage group than in the control group (29).

Finally, Sisti G et al. tried to assess whether NLR in the first trimester could be used as a predictive factor for the occurrence of HELLP syndrome but failed to show any significant results (30).

Few studies, however, have evaluated the link between Neutrophil-to-Lymphocyte ratio and SGA, and the results of these studies tend to differ from our own.

A case–control study of Levy et al. (31) were the first to evaluate NLR in the first trimester of pregnancy as a predictive marker of SGA. Their results were encouraging, with a significantly higher NLR in the SGA group than in the control group (3.0 vs. 2.6, *p* = 0.02 and 3.1 vs. 2.6, *p* = 0.03 for SGA < p10 and severe SGA < P3, respectively).

A second limited (*n* = 50) and retrospective study evaluated NLR and Platelet-to-Lymphocyte ratio (PLR) values in the first trimester of pregnancy, showing that NLR values were statistically higher in the intrauterine growth retardation group (32).

Finally, an Indian prospective observation study (*n* = 440) showed that a high NLR reflected a lower birth weight associated with a worse APGAR score at 5 min of life (33).

Finally, only Ersoy et al. (24) in their retrospective study of 408 patients found results similar to ours with a first-trimester NLR unrelated to SGA 2.90(+/−0.96) vs. 3.32(+/−1.36) *p* Value 0.22.

This difference in results can be explained by several factors. Our hypothesis is that uteroplacental insufficiency leads to hypoxia and increased oxidative stress, resulting in an inflammatory response. However, it is important to remember that around 60% of SGA neonates have no known etiology, and are therefore considered idiopathic (14), suggesting mechanisms of occurrence that have yet to be fully elucidated.

We can also assume that, given our population’s mean gestational age at delivery of 39 weeks’ gestation, the processes leading to SGA were not yet reflected in the hematological lineage at the time of blood sampling, and that the onset of SGA was delayed.

A fortiori, if the NLR elevation was already marked, it is likely that the course of the pregnancy would be affected by adverse events that could lead to earlier delivery of the fetus.

Regarding placentas, we found a significant difference in weight between the AGA and SGA groups. This is in line with the study by Kim et al. (34) which showed similar results to ours, i.e., a placental weight of 498 g (280–856) in the control group vs. 386 g (188–660) for the SGA group, with a *p*-value of <0.001. Concerning the BW/PW ratio, again the results were similar, with a BW/PW ratio value of 6.0 (3.9–11.1) vs. 6.1(3.1–10.3) for the SGA group, which was not significant.

This ratio is often used as an indicator of placental efficiency, providing an indication of the conditions encountered *in utero*.

One study showed that SGA infants had a higher BW/PW ratio than AGA infants between the end of the second and beginning of the third trimester, suggesting a decrease in placental function, but that this difference tended to fade as term was approached (35).

This was also demonstrated in a retrospective study of 18,386 IVF/ICSI pregnancies, which showed an increase in the placental ratio in pregnancies marked by SGA (36).

The effectiveness of the placenta in supporting fetal growth may vary according to the conditions encountered. When blood flow is restricted, the resulting hypoxemia and inflammatory response reduce the placenta’s effectiveness. In these circumstances, the availability of oxygen and nutrients will be used to maintain fetal survival *in utero* as far as possible.

The placenta will also adapt by decreasing the secretion of certain hormones such as glucocorticoids and IGFs in order to adapt the placental phenotype accordingly (37).

One of the strengths of our study is its prospective follow-up with complete and accurate data. One of its weaknesses is the relatively small sample size from a single hospital, which may have limited the ability to show an association between NLR and SGA. Another weakness is the confounding factors that make it difficult to isolate the inflammatory effect on fetal growth. Patients with a high-risk NIPT without aneuploidy will have a higher risk of SGA (38), but also patients with a history of SGA births (39). The SARS-CoV-2 pandemic, for which, in line with local recommendations, we did not systematically screen our patients unless they were symptomatic, and which can induce an inflammatory cascade leading to changes in hematological markers, as has been demonstrated (40), is also a confounding factor at this time.

## Conclusion

The neutrophil/lymphocyte ratio has been extensively researched as a marker of inflammatory response and, contrary to previous studies, we found no association between first-trimester NLR and SGA. Consequently, we do not recommend first-trimester NLR as a possible predictor of SGA.

## Data Availability

The original contributions presented in the study are included in the article/supplementary material, further inquiries can be directed to the corresponding author/s.
